# Glued suture-less peritoneum closure in laparoscopic inguinal hernia repair reduces acute postoperative pain

**DOI:** 10.1038/s41598-024-62364-w

**Published:** 2024-05-23

**Authors:** Michaël Huguenin-Dezot, Sarah Peisl, Evangelos Georgiou, Daniel Candinas, Guido Beldi, Christian Helbling, Joel Zindel

**Affiliations:** 1 Department of Visceral Surgery and Medicine, Inselspital, Bern University Hospital, University of Bern, Freiburgstrasse, 3010 Bern, Switzerland; 2 Department Für Allgemein- Und Viszeralchirurgie, Spital Linth, Gasterstrasse 25, 8730 Uznach, Switzerland

**Keywords:** Outcomes research, Pain

## Abstract

Inguinal hernia repair is performed more than 20 million times per annum, representing a significant health and economic burden. Over the last three decades, significant technical advances have started to reduce the invasiveness of these surgeries, which translated to better recovery and reduced costs. Here we bring forward an innovative surgical technique using a biodegradable cyanoacrylate glue instead of a traumatic suture to close the peritoneum, which is a highly innervated tissue layer, at the end of endoscopy hernia surgery. To test how this affects the invasiveness of hernia surgery, we conducted a cohort study. A total of 183 patients that underwent minimally invasive hernia repair, and the peritoneum was closed with either a conventional traumatic suture (*n* = 126, 68.9%) or our innovative approach using glue (*n* = 57, 31.1%). The proportion of patients experiencing acute pain after surgery was significantly reduced (36.8 vs. 54.0%, *p* = 0.032) by using *glue* instead of a *suture*. In accordance, the mean pain level was higher in the *suture* group (VAS = 1.5 vs. 1.3, *p* = 0.029) and more patients were still using painkillers (77.9 vs. 52.4%, *p* = 0.023). Furthermore, the rate of complications was not increased in the glue group. Using multivariate regressions, we identified that using a traumatic suture was an independent predictor of acute postoperative pain (OR 2.0, 95% CI 1.1–3.9, *p* = 0.042). In conclusion, suture-less glue closure of the peritoneum is innovative, safe, less painful, and possibly leads to enhanced recovery and decreased health costs.

## Introduction

Groin hernia is a prevalent disease with a life-time risk to require hernia surgery at least once of 3% for women and 27% for men. This results in over 20 million hernia surgeries performed annually. Over the last three decades^1^ the minimally-invasive approaches to hernia repair such as laparoscopic and robotic transabdominal preperitoneal (TAPP) and totally extraperitoneal (TEP) hernia repair, have gained in importance compared to open repair—such as the Lichtenstein technique—and become the gold standard^2^. In comparison to open surgery, the minimally invasive techniques reduce acute postoperative pain and thus allow a quicker return to work^3,4^. Since most hernia patients are of working age^5^, return to work is an important outcome measure after hernia surgery. The mean absence from work after minimally invasive inguinal hernia repair is of 6.4–7 days^4,6^. While this is better than in open repair (11 days^4^), it still cumulates in a significant socioeconomic burden given the high prevalence of the problem.

In addition, about 11% of patients suffer chronic pain after mesh-based inguinal hernia repair^7^. Non-penetrative mesh fixation had already been shown to be safe by Kukleta et al. in 2012^8^, to decrease postoperative pain^9^ in randomized controlled trials^10,11^ and is now generally preferred over penetrative tacker fixation^5^. During TAPP procedure, in addition to the mesh implantation, the peritoneal flap needs to be closed which is routinely performed using a penetrative (resorbable) suture. Small case series brought forward that gluing may be a safe and fast alternative to suturing^12–15^. However, It remains unclear whether gluing instead of suturing brings a clinical benefit.

We hypothesize that using glue to close the peritoneal flap reduces the acute postoperative pain. To test this hypothesis, we performed a before-after cohort study after implementation of a glue applicator (Glutack) to close the peritoneal flap during TAPP. We find that atraumatic peritoneal flap closure with glue instead of a suture reduces acute postoperative pain. There was no effect on the safety and secondary outcomes such as complications, chronic postoperative pain or recurrence 1 year after laparoscopic inguinal hernia repair.

## Materials and methods

### Study design and data collection

**Figure 1:**
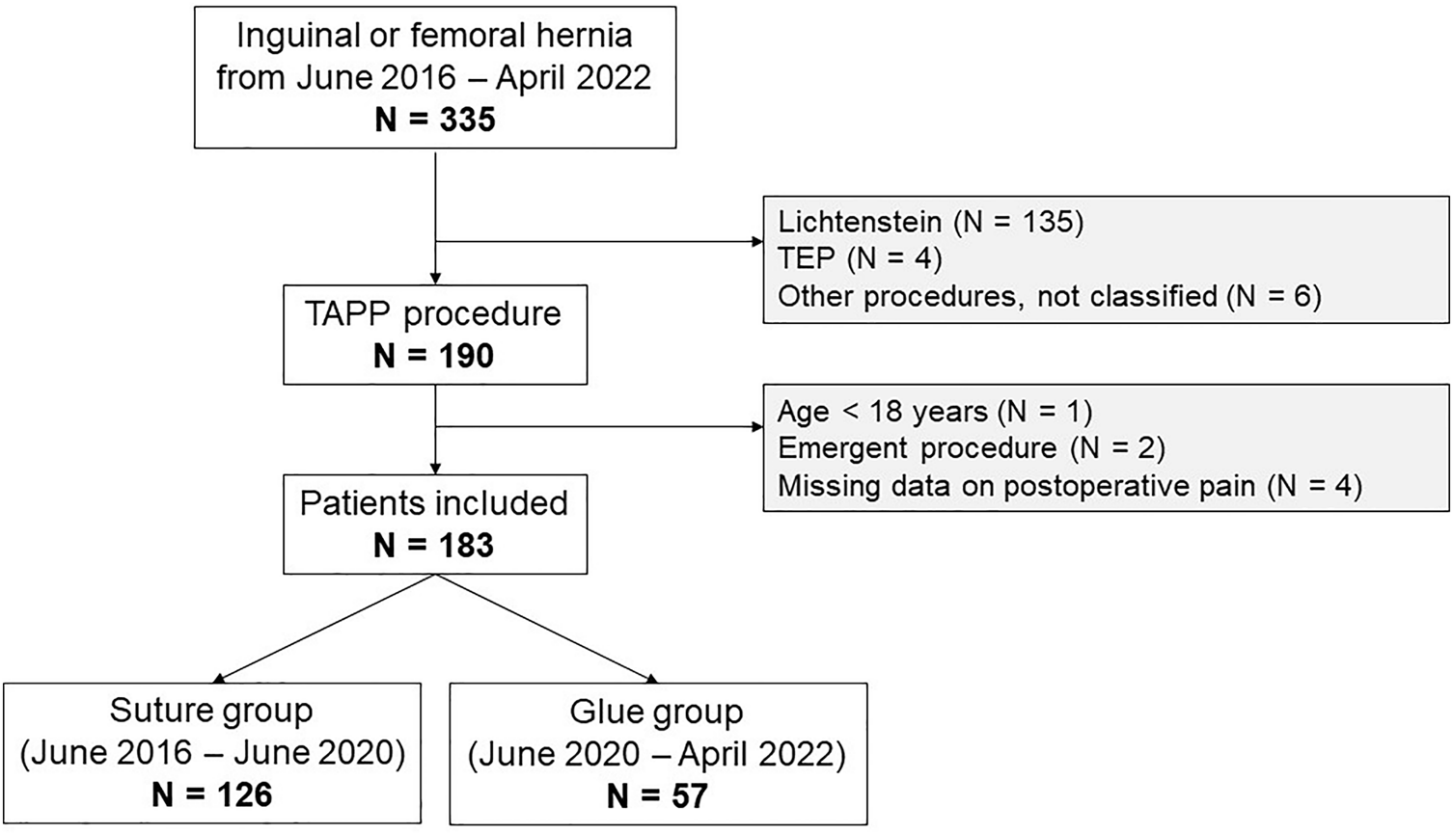
Flowchart of patient selection.

To investigate the impact of suture-less peritoneal flap closure on postoperative pain we conducted a retrospective before-after cohort study. The data of this study were prospectively collected using the Herniamed quality control study^16^. Herniamed is an international, internet-based quality assurance registry in which data on hernia surgery can be registered by hospitals and individual surgeons. All patients signed a consent form before enrollment. The, data collected on the Herniamed registry include patient demographics, health status, details on hernia and surgical procedure as well as follow-up data (up to ten years). From the Herniamed registry, all patients operated by one surgeon (CH) were selected. All adult patients that underwent elective inguinal or femoral hernia repair using the TAPP technique between June 2016 (registration to Herniamed) and April 2022 were included. Exclusion criteria were age under 18 years and if the repair was done as an emergency procedure (Fig. 1). All patients registered for this study gave their informed consent in writing (Fig. S1). The study was approved by the cantonal ethics committee (Ethikkommission Ostschweiz, Project ID 2016–00,123). All methods were performed in accordance with the relevant guidelines and regulations.

The primary outcome of acute postoperative pain was systematically assessed with a simple yes/no question at 7–10 days post-surgery. The investigator (CH) was not blinded. In addition, pain was also quantified on a Visual Analogue Scale (VAS) (0 representing no pain and 10 corresponding to the worst possible pain) at the same time. Additional outcomes were collected in the study according the Herniamed protocol (Supplementary Table S1). Follow-up data (chronic pain and symptomatic recurrence) were collected after one year, using standardised questionnaire sent per Mail with a stamped reply envelope.

### Operative technique

All study patients were operated by the same surgeon (CH). This surgeon had 19 years of experience in laparoscopic hernia surgery prior to begin of the study. This is more than necessary to reach a steady state in the learning curve for this procedure^17^. The surgical indication and procedure were carried out according to current standard of care recommendations^5,18,19^. In June 2020, the surgical routine of peritoneal flap closure was altered from using a suture to using glue without exception. Therefore, patients that received surgery before June 2020 were exposed to suturing and thus comprise the *suture* group, and the patients operated after June 2020 to April 2022 were exposed to gluing and therefore comprise the *glue* group. All patients were operated under general anaesthesia with intravenous disoprivan, remifentanil and fentanyl in supine position. No transversus abdominis plane block was performed.

A camera trocar was placed in midline 2–3 cm over the umbilicus (12 mm). After establishing a pneumoperitoneum of 12 mmHg two additional trocars (5 mm in the left, 12 mm in the right mid-abdomen) were inserted. Then, the peritoneum was incised about 6 cm cranial to the inner inguinal ring from lateral to medial. The preperitoneal space was dissected and the hernia sac reduced. Then, after a control of haemostasis, the mesh (Polypropelene Lightweight Monofilament Mesh of 10 × 15 cm, Parietene™ flat lightweight) was inserted and fixed with 8–10 single applications of Glubran®. In the *Suture* group, the peritoneal flap was then closed with a running suture using a resorbable barbed monofilament suture (V-Loc™). In the *glue* group, the peritoneum was closed using a cyanoacrylate glue [Glubran® 2 (N-butyl cyanoacrylate metacryloxysulfolane, NBCA-MS)] applied with 10–15 applications of Glutack® (Supplementary Video 1). Then, the trocars were removed under visual control and the fascia was sutured for the 12 mm trocar using resorbable back stitch. Postoperatively, all patients received Ibuprofen (400 mg p.o. qid) and Paracetamol (1 g p.o. qid). In patients with renal insufficiency, Ibuprofen was replaced by metamizole (1000 mg p.o. qid).

### Statistical analysis

The baseline characteristics were described hernia-wise and patient-wise. For patient-wise comparison, the following criteria were applied for all patients with bilateral hernia: (i) hernia size was defined by the size of the larger hernia. (ii) If the patient had more than one hernia, it was considered as a combined hernia irrespective of location. (iii) Recurrent hernia was defined by having at least one recurring hernia.

Dichotomous data were reported as number and proportion, and continuous data as mean and standard deviation (SD). Categorical data were compared with Pearson's chi-squared or Fisher’s Exact Test. Continuous variables were compared by parametic tests (t-test) when normally distributed or by non-parametric tests (Wilcoxon) when non-normally distributed.

To adjust for potential confounding, a multivariate regression model was used. First univariate logistic regression was performed to explore the unadjusted association between each patient- and hernia-related variable and the primary outcome. Next, a multivariate regression model was fit with all variables statistically relevant in the unadjusted model. A two-sided level of significance of 0.05 was used for all analyses. All statistical analyses were performed using R statistical environment^20^. The underlying raw data and R scripts necessary to reproduce all figures and tables are available in the Supplementary Materials.

## Results

### Patient demographics and baseline characteristics

A total of 183 patients were enrolled with N = 126 in the *suture* and N = 57 in the *glue* group (Fig. 1). Demographic variables age (*p* = 0.9), BMI (*p* = 0.3), sex (*p* = 0.7) and ASA-score (*p* = 0.2) were not significantly different between groups (Table 1). However, the frequency of patients with preoperative pain was significantly higher in the *suture* group (56 vs 37%, *p* = 0.019) when compared with the *glue* group.

**Table 1:** Patient demographics and preoperative pain.

Variable	N	Suture, N = 126^1^	Glue, N = 57^1^	*p*-value^2^
Age	183	55 (15)	55 (14)	0.9
BMI	183	24.99 (2.66)	25.52 (3.10)	0.3
Sex	183			0.7
Female		11/126 (9%)	6/57 (11%)	
Male		115/126 (91%)	51/57 (89%)	
ASA Score	183			0.2
1		99/126 (79%)	40/57 (70%)	
2		27/126 (21%)	16/57 (28%)	
3		0/126 (0%)	1/57 (2%)	
Presence of preoperative pain	183	70/126 (56%)	21/57 (37%)	0.019

^1^Mean (SD); n/N (%).

^2^Wilcoxon rank sum test; Pearson's chi-squared test; Fisher's exact test.

### Hernia characteristics

Next, we asked whether the hernia characteristics between the two groups were different (Table 2). In this study, a total of N = 258 hernias were operated (N = 175 in the *suture*, N = 83 in the *glue* group) in N = 183 patients (Fig. 1). There were more medial (31 vs. 18%) but less combined inguinal hernias (17 vs. 33%) in the *suture* group (*p* = 0.012) as defined by the Aachen classification (Table 2)^21^. Looking at size distribution, most hernias showed a maximal diameter between 1.5 and 3 cm in both groups, representing a Grade II inguinal hernia. There were slightly more Grade I hernias in the *suture* group (15 vs. 8.4%) and fewer Grade III (20 vs. 39%) (Table 2). The proportion of primary (vs. recurrent) hernias was the same in both groups. In terms of size, the *suture* group showed a significantly lower percentage of larger hernias (20 vs. 39% Hernia Grad III, *p* = 0.005). No significant difference in the primary hernia rate was found between two groups (93 vs. 95%) (Table 2).

**Table 2:** Hernia characteristics (per hernia).

Variable	N	Suture, N = 175^1^	Glue, N = 83^1^	*p*-value^2^
Aachen Classification	258			0.012
Combined		29 / 175 (17%)	27 / 83 (33%)	
Femoral		3 / 175 (2%)	2 / 83 (2%)	
Lateral		88 / 175 (50%)	39 / 83 (47%)	
Medial		55 / 175 (31%)	15 / 83 (18%)	
Size	258			0.005
I (< 1.5 cm)		26 / 175 (15%)	7 / 83 (8%)	
II (1.5-3 cm)		114 / 175 (65%)	44 / 83 (53%)	
III (> 3 cm)		35 / 175 (20%)	32 / 83 (39%)	
Primary Hernia	258	162 / 175 (93%)	79 / 83 (95%)	0.43

^1^n / N (%); Mean (SD).

^2^Fisher's exact test; Pearson's Chi-squared test; Wilcoxon rank sum test.

To compare the hernia characteristics per patient, the data of patients with bilateral hernia repair was processed as described in the methods section. Overall, the two treatment groups are comparable (Table 3). Notably, there was a significant difference in hernia size between the groups with a higher rate of size I (9 vs. 2%) and size II (65 vs. 56%) but less size III hernias (26 vs. 42%) in the *suture* group (*p* = 0.043) (Table 3).

**Table 3:** Hernia characteristics (per patient).

Variable	N	Suture, N = 126^1^	Glue, N = 57^1^	*p*-value^2^
Aachen Classification	183			0.6
Combined		62 / 126 (48%)	33 / 57 (58%)	
Femoral		3 / 126 (2%)	0 / 57 (0%)	
Lateral		48 / 126 (38%)	18 / 57 (32%)	
Medial		13 / 126 (10%)	6 / 57 (10%)	
Side	183			0.4
Bilateral		49 / 126 (39%)	26 / 57 (46%)	
Unilateral		77 / 126 (61%)	31 / 57 (54%)	
Size	183			0.043
I (< 1.5 cm)		11 / 126 (9%)	1 / 57 (2%)	
II (1.5-3 cm)		82 / 126 (65%)	32 / 57 (56%)	
III (> 3 cm)		33 / 126 (26%)	24 / 57 (42%)	
Primary Hernia	183	117 / 126 (93%)	54 / 57 (95%)	0.8

^1^Mean (SD); n / N (%).

^2^Wilcoxon rank sum test; Pearson's chi-squared test; Fisher's exact test.

### Postoperative pain

**Figure 2:**
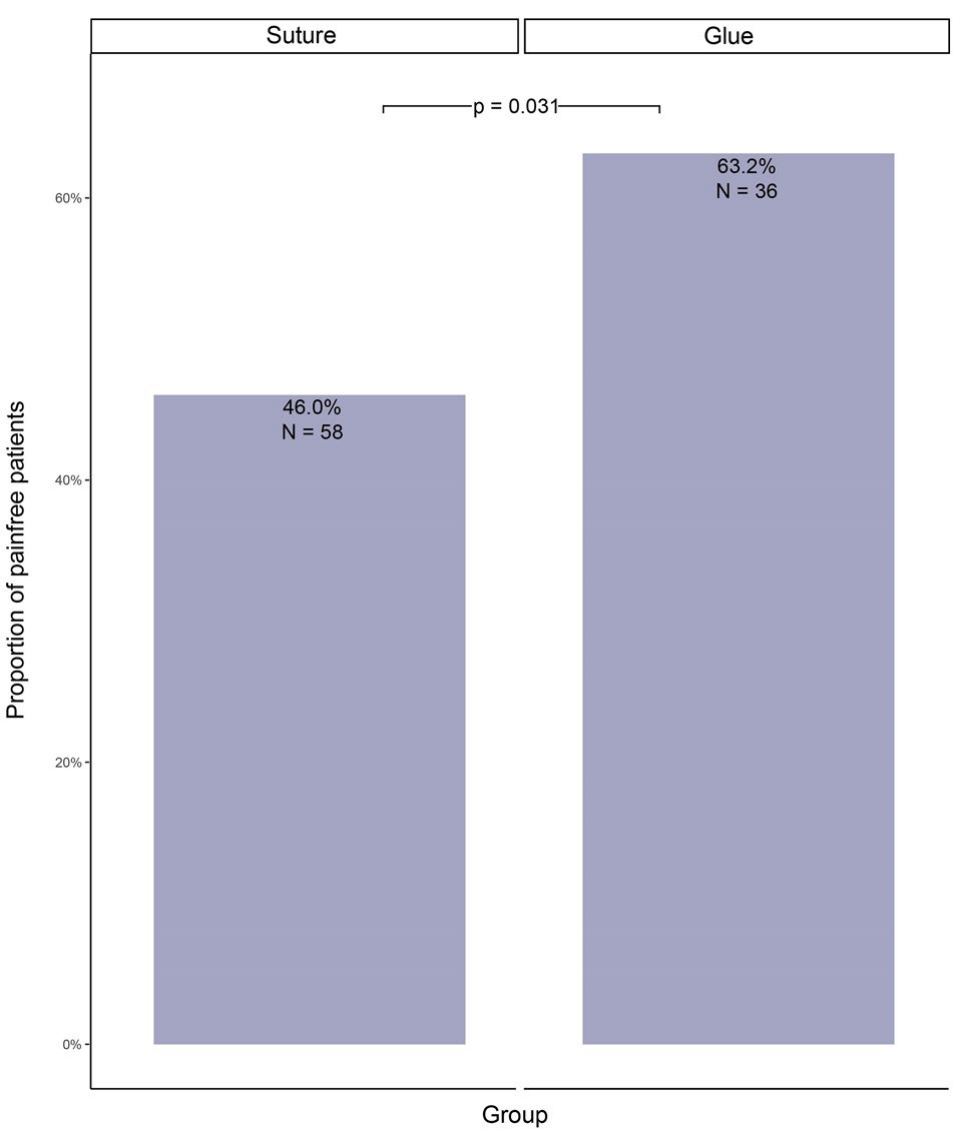
Painfree patients within 10 days of operation.

The rate of patients feeling pain 7–10 days after operation is significantly higher in the *suture* group when compared with the *glue* group (respectively 54 vs 37%, *p* = 0.032) (Table 4, Fig. 2). Congruently, the pain score (VAS = 1.50 vs. 1.31, *p* = 0.029) and the rate of patients still depending on painkillers (78 vs. 52%, *p* = 0.023) were significantly increased in the *suture* group (Table 4).

**Table 4:** Postoperative follow-up data.

Variable	N	Suture, N = 126^1^	Glue, N = 57^1^	*p*-value^2^
Pain after 7–10 days	183	68 / 126 (54%)	21 / 57 (37%)	**0.032**
Degree of pain after 7–10 days	183	1.34 (1.50)	0.88 (1.31)	**0.029**
Need for Analgesia after 7–10 days	89	53 / 68 (78%)	11 / 21 (52%)	**0.023**
Unknown		58	36	
Duration of surgery [minutes]	183	67 (24)	71 (27)	0.4
Complications (without pain)	183	8 / 126 (6.3%)	0 / 57 (0%)	0.059
Intraoperative Complications		3 / 126 (2.4%)	0 / 57 (0%)	0.6
Postoperative Complications		4 / 126 (3.2%)	0 / 57 (0%)	0.3
Postoperative Hematoma		3 / 126 (2.4%)	0 / 57 (0%)	0.6
Postoperative Seroma		1 / 126 (0.8%)	0 / 57 (0%)	> 0.9

Follow-up 1 year postoperative				
Pain	132	11 / 101 (11%)	4 / 31 (13%)	0.8
Unknown		25	26	
Pain at rest	132	3 / 101 (3.0%)	1 / 31 (3.2%)	> 0.9
Unknown		25	26	
Pain by physical Stress	132	10 / 101 (9.9%)	4 / 31 (13%)	0.7
Unknown		25	26	
Dysesthesia	132	2 / 101 (2.0%)	0 / 31 (0%)	> 0.9
Unknown		25	26	
Recurrence of Hernia	92	1 / 71 (1.4%)	0 / 21 (0%)	> 0.9
Unknown		55	36	
Reason for Break Off of Follow-up	163			0.4
No break off		101 / 126 (80%)	31 / 37 (84%)	
Patient moved		1 / 126 (0.8%)	1 / 37 (2.7%)	
Other reasons for break off		24 / 126 (19%)	5 / 37 (14%)	
Unknown		0	20	

^1^Mean (SD); n / N (%).

^2^Wilcoxon rank sum test; Pearson's Chi-squared test; Fisher's exact test.

Statistically significant values are in bold.

In our univariate analysis, we identified 5 predictors for acute postoperative pain (7–10 days post-surgery): peritoneal flap suturing (OR 2.01, *p* = 0.031), younger age (OR 1.03, *p* = 0.002), presence of preoperative pain (OR 1.81, *p* = 0.046), female sex (OR 3.85, *p* = 0.014) and small hernia size (size II OR 2.9, size III OR 4.77, *p* = 0.049) (Table 5).

**Table 5:** Painfree 7–10 days after operation (univariate regression model).

Variable	N	Event N	OR^1^	95% CI^1^	p-value
Group	183	94			**0.031**
Suture			–	–	
Glue			2.01	1.07, 3.86	
Age	183	94	1.03	1.01, 1.06	**0.002**
BMI	183	94	1.05	0.95, 1.17	0.34
Sex	183	94			**0.014**
Female			–	–	
Male			3.85	1.30, 14.1	
ASA score	183	94	1.38	0.72, 2.71	0.33
Previous operation	183	94			0.52
No			–	–	
Yes			1.29	0.59, 2.89	
Preoperative pain	183	94			**0.046**
Yes			–	–	
No			1.81	1.01, 3.27	
Aachen classification	183	94			0.20
Combined			–	–	
Femoral			0.00		
Lateral			1.00	0.53, 1.87	
Medial			1.29	0.48, 3.60	
Side	183	94			0.89
Bilateral			–	–	
Unilateral			0.96	0.53, 1.73	
Size	183	94			**0.049**
I (< 1.5 cm)			–	–	
II (1.5-3 cm)			2.90	0.82, 13.6	
III (> 3 cm)			4.77	1.27, 23.3	
Primary hernia	183	94			0.92
No			–	–	
Yes			1.06	0.32, 3.51	
Duration of surgery [minutes]	183	94	0.99	0.98, 1.00	0.21

^1^OR = Odds Ratio, CI = Confidence Interval.

Statistically significant values are in bold.

When adjusted in a multivariate regression model, the three independent predictors of acute postoperative pain (7–10 days post-surgery) were as follows. Peritoneal flap suturing (OR 2.07, *p* = 0.042), younger age (OR 1.03, *p* = 0.004) and female sex (OR 4.57, *p* = 0.015) (Table 6).

**Table 6:** Painfree 7–10 days after operation (multivariate regression model).

Variable	OR^1^	95% CI^1^	*p*-value
Group			**0.042**
Suture	–	–	
Glue	2.07	1.03, 4.26	
Age	1.03	1.01, 1.06	**0.004**
Sex			**0.015**
Female	–	–	
Male	4.57	1.33, 19.0	
Preoperative Pain			0.66
Yes	–	–	
No	1.16	0.60, 2.22	
Size			0.79
I (< 1.5 cm)	–	–	
II (1.5-3 cm)	1.45	0.37, 7.20	
III (> 3 cm)	1.67	0.38, 8.89	

^1^OR = Odds Ratio, CI = Confidence Interval.

Statistically significant values are in bold.

Unlike previous reports, we did not observe a significant decrease in operation time in the *suture* group (67 min) when compared with the *glue* group (71 min, *p* > 0.05), and the intra- and early postoperative complication rate were comparable in both groups (respectively 6.3 vs 0%, *p* = 0.059).

Furthermore, the two approaches resulted in comparable outcomes after 1 year follow-up. The rate of patients experiencing chronic pain was comparable found between the *suture* and *glue* groups (respectively 11 vs 13%, *p* > 0.05) whether for pain at rest (3 vs 3.2%, *p* > 0.05) or pain during physical stress (9.9 vs 13%, *p* > 0.05). As for the dysesthesias experienced one year postoperatively, no significant difference were found for hernia recurrence rate after 1 year (Table 4).

## Discussion

Here we present a comparative cohort study, supporting the introduction of an innovative concept: utilizing a glue applicator instead of a suture to close the peritoneal flap after TAPP. Our findings demonstrate that this less invasive approach significantly reduces acute pain levels experienced 7–10 days post-surgery. Because inguinal hernia repair is so frequently performed^22,23^ and often in patients of working age^24^, this small improvement in care amounts to a huge beneficial effect for health care systems.

Laparoscopic suturing has a relatively long learning curve, which ideally should first be practiced outside the operating theatre in a specific box-training before it can be performed on patients^25–27^. Incremental technological support (e.g. 3D screens, virtual reality training) attempts to make the surgeon's job easier^28–30^. Here we bring forward yet another approach: the use of a glue applicator, which is fast, intuitive, and safe for the patient^31^.

The use of glue in the abdominal cavity is still subject to intense debate. The acrylic adhesive utilized in this study (Glubran2®) deviates from the N-butyl-2-cyanoacrylate (NBCA) monomer present in Histoacryl® due to the inclusion of metacryloxysulfolane (MS). This addition, acting as a comonomer, modifies its chemical composition and confers anti-inflammatory properties, along with changing its viscosity among other characteristics^32^. Glue mesh-fixation has been evaluated in a prospective manner and gluing the mesh in place, results in a significant reduction of chronic pain and haematoma rate, when compared to mechanical fixation (high level of evidence)^33,34^. Its use has also been described in the context of incisional hernia repair by Laparoscopic intraperitoneal onlay mesh (IPOM)^31^. However, these studies investigate the effect on mesh fixation. Only small retrospective, non-comparative studies reported its implementation in the context of peritoneal closure^12,35^. In their 2016 publication, Köckerling et al. report a postoperative bleeding occurrence of about 1.5% after endoscopic hernia repair^36^. While the current cohort is clearly underpowered to detect a significant difference of such a rare event, we argue on a conceptual level that using glue instead of a suture might reduce the bleeding risk.

Furthermore, glue fixation of the peritoneal flap did not increase hernia recurrence rate. This statement is limited, because the current cohort is neither sufficiently powered nor followed up long enough for this specific question. However, recent experimental studies conducted by Lesch et al. on biomechanical constraints in abdominal reconstruction suggest that stability primarily emerges during the period of plastic deformation. This process appears to occur mainly during the initial mechanical stresses^37^.

While not insignificant, the closure of the peritoneum appears to have a secondary impact on biomechanical stability following mesh hernia repair^38^. Overall, we conclude that glue closure of the peritoneal flap after TAPP is safe. The research conducted by Kallinowski et al. assigns it a value as a term rather than a factor within the equation used to calculate the GRIP^39^ value, assessing resistance of the Mesh-reconstruction (Gained/Critical Resistance to Impacts related to Pressure). Undoubtedly, this is a hot topic, and the principles of biomechanically calculated repair (BCR), which have shown promising outcomes for incisional hernias^40^ should be carefully considered in patient-tailored inguinal hernia surgery.

Our data do not allow us to infer on the exact pathophysiological reasons why post-surgical pain is decreased after gluing. However, some studies seem to suggest that sutures in the abdominal wall may create ischemic areas in the muscle, triggering inflammation and therefore pain^41^. Importantly, the beneficial effect of gluing was lost after one year. The overall percentage of patients with chronic pain (defined as lasting more than 3 months after the operation) in this study was 11% und therefore well comparable with the numbers reported in the literature^5^. As the suture used is absorbable and has been resorbed after this time (V-Loc™ resorption after 180 days^42^), we assume that it contributes to the difference of initial acute postoperative pain but not the chronic pain. Interestingly, the beneficial effect of gluing might be lost in the elderly as we observed that older ager is an independent protective factor against acute postoperative pain. This difference is in line with the current literature, in the context of decreased function of the nociceptive sensation and an increased pain perception threshold in the elderly^43,44^ and possibly also generational differences in pain experience^41^. Conversely—although the study is clearly underpowered to back this up—the positive effect of peritoneal gluing may be even more pronounced in women because women have an independent propensity to experience more pain after inguinal hernia surgery. This effect, of unknown pathophysiology and probably multivariate etiology has already been observed and discussed in several studies whose results tended to demonstrate the same effect^45–47^. Taken together, we bring forward that gluing is less painful than suturing most likely because it is less traumatic, an effect that may be particularly desirable in young and female patients with pre-operative pain.

The novelty of our study is that we use glue for the closure of the peritoneum, and not only to fixate the mesh and that we compare this technique with an established approach. This shows a remarkable beneficial effect of gluing over suturing. But because this study is limited by the cohort size and its retrospective non-blinded nature, the current data alone may not warrant a recommendation of gluing over suturing just yet. In addition, the study suffers from missing data regarding the analgesic use and follow-up data and some, presumably asymptomatic, patients were lost to follow-up after one year. A further limitation of this study is that the change in surgical technique (suture vs glue) coincides in time with a change of the policy makers in Switzerland that decreed that outpatient surgery should be prioritized over inpatient surgery for unilateral inguinal hernia repair^48^. This may be a systematic bias in this study that cannot be addressed by study design, and it was also the reason why neither the length of hospital stay, nor the treatment costs were compared in this cohort. Despite these limitations, the current study is the first to systematically compare suturing versus gluing the peritoneal flap after TAPP procedure and clearly provides evidence that the technique is safe and might be superior in terms of acute pain. Clearly, further register based studies or even a randomized controlled trial is necessary to infer on the true potential of this surgical technique.

## Conclusion

This study provides solid evidence that the closure of the peritoneal flap after TAPP with cyanoacrylate glue instead of a suture is less painful and at least equally safe. This innovative surgical technique, which has not yet been comparatively studied in the current literature, may be preferable over suturing, especially in women, in the young, and in patients with pre-operative pain.

### Supplementary Information

Supplementary Information 1.

Supplementary Video 1.

Supplementary Information 2.

## Data Availability

All data generated or analysed during this study are included in this published article (and its Supplementary Information files). Further inquiries can be directed to the corresponding author/s.
