# Supplementary material for: Glued suture-less peritoneum closure in laparoscopic inguinal hernia repair reduces acute postoperative pain
Source: Sci Rep. 2024 May 23;14:11786. doi: 10.1038/s41598-024-62364-w (PMC11116422; doi:10.1038/s41598-024-62364-w)
Supplement: Supplementary file 1 — Supplementary Information 1. [file 41598_2024_62364_MOESM1_ESM.docx]

| Basic data | \| Patient ID \| \| --- \| \| Case ID \| \| Name \| \| Surname \| \| Date of birth \| \| Sex \| \| Height \| \| Weight \| \| Surgery \| \| Family doctor \| \| Adress \| |
| --- | --- | --- | --- | --- | --- | --- | --- | --- | --- | --- | --- | --- |
| Risk factor | \| COPD \| \| --- \| \| Immunsuppression \| \| Coagulation disorder \| \| Liver cirrhosis \| \| Diabetes \| \| Cortisone \| \| Vitamin K antagonists oder NOACs \| \| Aortic aneurysm \| \| Nicotine abuse \| \| Coumarin derivatives/Quick-INR abnormal \| \| ASA \| \| Pre-surgery \| \| Preoperative pain (VAS) \| \| Surgeon \| \| Outpatient/inpatient \| \| Urgency (elective/emergency) \| \| Reduction of the hernia sac \| \| Incarceration \| \| Intestinal resection \| \| Surgery date \| \| Type of anaesthesia \| \| OP duration (min) \| \| Antibiosis \| \| Surgery primary/recurrent \| \| Aachen classifikation \| \| EHS classifikation \| \| Hernia size (cm) \| \| Access \| \| Surgical method \| \| Direct suture? \| \| Spermatic cord lipoma/Lipoma of the round ligament of uterus \| \| Reduction of lipoma? \| |

**List of data collected in Herniamed**

| Mesh | \| Mesh type \| \| --- \| \| Mesh size \| \| Fixation ? \| \| Stitching ? \| \| Stapler ? \| \| Glue ? \| \| Drainage \| |
| --- | --- | --- | --- | --- | --- | --- | --- | --- |
| Complications | Intraoperative complication (bleeding/injury)  Post-op complications (post-operative bleeding, intestinal injury, suture insufficiency, wound healing disorder, seroma, infection, ileus)  Reoperation |
| Specific complications | \| Nerve lesion \| \| --- \| \| Dysaesthesia \| \| Other \| |
| General complications | \| Fever, gastritis/ulcer \| \| --- \| \| Pleural effusion \| \| Heart failure \| \| Myocardial infarction \| \| UTI \| \| Thrombosis \| \| Pneumonia \| \| Hypertensive crisis \| \| Renal failure \| \| Diarrhoea \| \| Pulmonary embolism \| \| COPD/asthma \| \| CHD \| \| Patient deceased \| \| Other \| \|  \| |

Supplementary Table S1
