# Supplementary material for: Glued suture-less peritoneum closure in laparoscopic inguinal hernia repair reduces acute postoperative pain
Source: Sci Rep. 2024 May 23;14:11786. doi: 10.1038/s41598-024-62364-w (PMC11116422; doi:10.1038/s41598-024-62364-w)
Supplement: Supplementary file 3 — Supplementary Information 2. [file 41598_2024_62364_MOESM3_ESM.docx]

**Supplementary Video 1**: *A: The edges of both peritoneal flaps are glued together using interrupted glue application. The surgical thread visible is a positioning aid placed on the mesh prior to implantation and it is independent of the glue closure of the peritoneum. B: After each glue application, the flap is held in position for a few seconds. C: In addition, smaller peritoneal defects are closed using the same method. D: Final result.*
